# Magnetic Apposition across the Epiglottis: Radiographic and Operative Correlation of a Rare Hypopharyngeal Foreign Body

**DOI:** 10.1155/2020/3245634

**Published:** 2020-02-03

**Authors:** Yeli Pi, Shilpa Radhakrishnan, Yaser Alrajhi, Ravi Bhargava

**Affiliations:** ^1^Department of Radiology and Diagnostic Imaging, University of Alberta, Edmonton, Alberta, Canada; ^2^Department of Otolaryngology Head and Neck Surgery, University of Alberta, Edmonton, Alberta, Canada

## Abstract

*Background and Aim*. Rare-earth magnet ingestions are a subset of foreign body ingestions and can result in significant morbidity secondary to pressure necrosis. These magnets are best visualized radiographically, typically located in the gastrointestinal tract. However, unusual locations of magnetic adherence may include the hypopharynx along the epiglottis, where only 2 previous cases have been reported. Clinicians should be aware of the potential dangers of rare-earth magnet ingestion and consider atypical locations of attachment in the appropriate clinical setting. *Case Presentation*. We present an interesting case of a fourteen-year-old female patient who presents with witnessed ingestion of multiple rare-earth magnets. Soft-tissue neck radiographs demonstrate two adjacent rounded radiopaque densities in the hypopharynx. Intraoperative images confirmed the radiographic findings and identified two magnetic balls stuck along the dorsal and ventral aspect of the epiglottis without evidence of pressure necrosis.

**Conclusion:**

This is the first published case of magnetic foreign body adherence to the epiglottis in the Radiology literature. Awareness and recognition of the unique radiographic findings of this rare entity can help clinicians streamline timely management.

## 1. Introduction

Foreign body ingestion/aspiration is a common event in children and adolescents, accounting for over 60,000 cases annually in the United States [1]. While coins, balls, and pearls are some of the more commonly ingested/aspirated items [2], clinicians must maintain a high degree of suspicion for magnets and batteries, due to their potential for serious and even fatal sequelae. A particularly dangerous subset of magnets is small rare-earth magnets such as neodymium magnets, which are substantially stronger than traditional ferrite magnets [3, 4]. Ingestion of multiple of these “supermagnets” is of special concern, as they can attract through intestinal walls, resulting in pressure necrosis, fistulization, and bowel perforation [5–7].

Numerous cases of rare-earth magnet ingestion have been reported in the literature [4–8]. However, hypopharyngeal magnetic foreign body aspirations are less commonly described.

We present a rare case of epiglottic magnetic foreign bodies in a 14-year-old patient. To our knowledge, this is the third case of magnetic foreign body adherence to the epiglottis in the current literature and the first published case in the Radiology literature.

## 2. Case Presentation

A 14-year-old, immunized, previously healthy female presented to our institution, a large tertiary pediatric center, with the chief complaint of “foreign bodies.” As per triage note timed 12:23 hours, the patient described having “swallowed two magnetic balls…[and] thinks they are stuck in the throat.” The patient was in no acute distress, and her vitals were stable including a normal blood pressure and 97% oxygen saturation on room air. She was assigned category 3 on the Canadian Triage and Acuity Score (CTAS), an “Urgent” rating that recommends time to physician evaluation less than 30 minutes [9].

**Figure 1:**
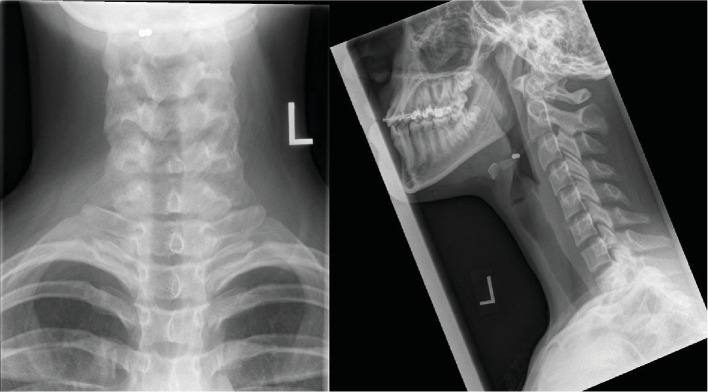
AP and lateral images of the neck demonstrate two adjacent rounded 3 mm metallic foreign bodies at the C2-3 level eccentric to the right.

The patient was assessed by a pediatric emergency physician within an appropriate timeframe and sent for further evaluation. AP and lateral nasopharyngeal radiographs were obtained at 13:17 hours, which demonstrated two adjacent round 3 mm metallic densities at the C2-3 level, slightly eccentric to the right (Figure 1). The location suggested that the magnets were potentially stuck to the epiglottis. A single additional similar-appearing metallic object was seen in the right lower quadrant on abdominal radiograph (image not shown).

**Figure 2:**
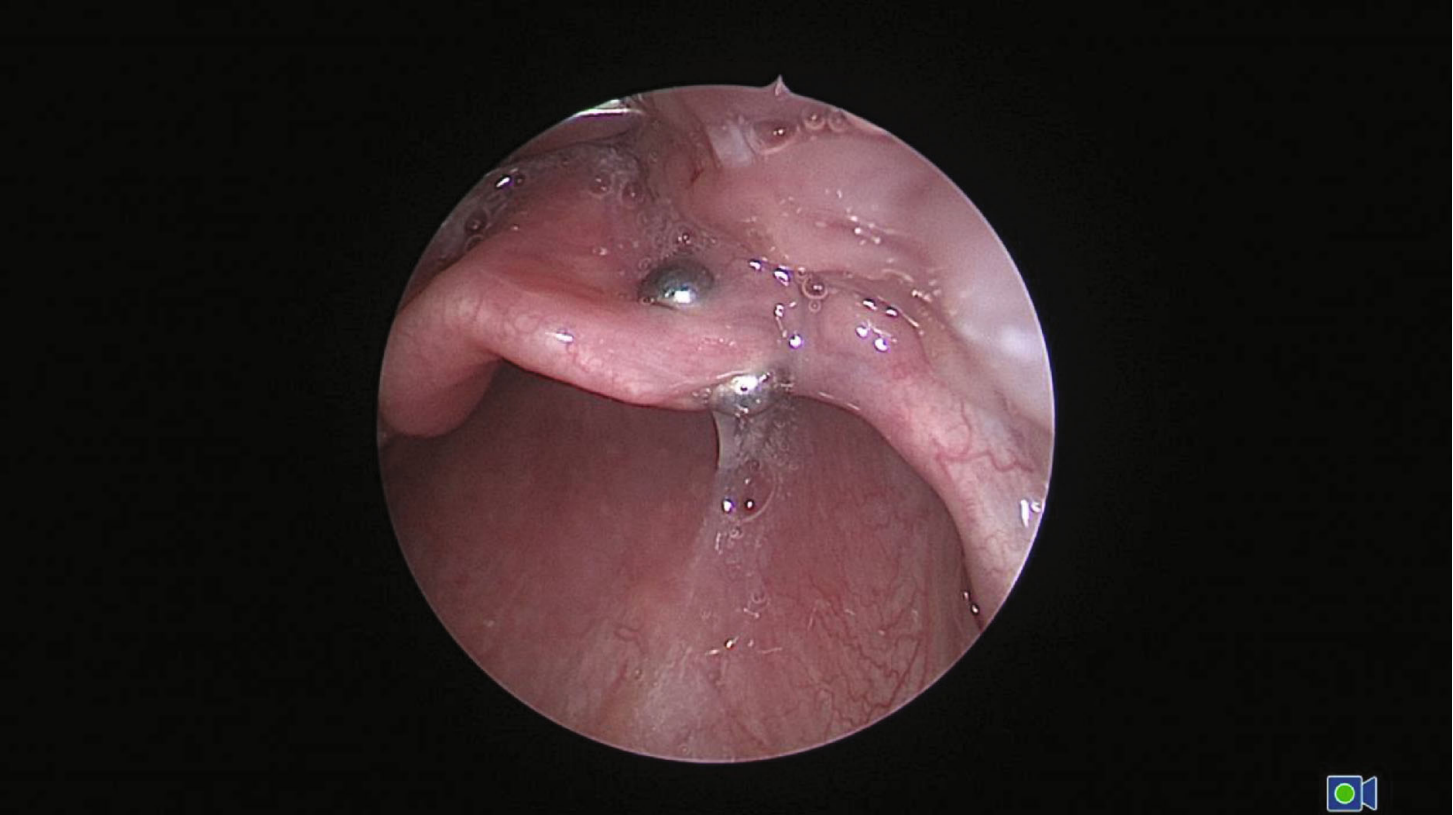
Intraoperative image demonstrates ball magnets along the ventral and dorsal side of the epiglottis, corresponding to radiographic findings.

The findings were urgently relayed to the referring clinician, as well as the pediatric otolaryngology service. Shortly after, the patient was brought to the operating room by 14:23 hours. Under laryngoscopy, two tiny spherical magnets were visualized along the ventral and dorsal surface of the epiglottis eccentrically to the right, corresponding to the radiographic findings (Figure 2). The epiglottis appeared slightly edematous, with no obvious evidence of pressure necrosis. The magnets were retrieved simultaneously via forceps. The patient was stable throughout the entire procedure and was discharged home after two hours of postprocedural monitoring. The single gastrointestinal magnet was treated conservatively and allowed to pass with bowel movements.

## 3. Discussion

Foreign body aspiration/ingestion events may present with a wide variety of symptoms ranging from completely asymptomatic to gagging, coughing, vomiting, or severe respiratory distress [10–13]. Thus, a chief complaint of magnetic foreign body ingestion/aspiration should be triaged as high acuity, for instance a CTAS category 2 (Emergent; time to physician assessment less than 15 minutes) or even CTAS category 1 (Resuscitative; immediate physician assessment) in the appropriate clinical setting [9]. This history should also trigger a detailed investigation including a thorough history, physical examination, and imaging.

As prior studies have indicated, the primary means for investigating suspected foreign body is conventional radiography, including views of the neck, chest, and abdomen [11, 14, 15]. It is crucial for clinicians and radiologists to complete the radiographic evaluation, as the absence of a foreign body at one location does not exclude upstream or downstream culprits. Conversely, if a foreign object is identified (such as in our patient), we still encourage completing additional imaging to avoid the satisfaction of search and prevent undue morbidity due to delayed diagnosis. Cross-sectional imaging may be considered as the next step of investigation, especially if there is strong clinical concern for a nonradiopaque foreign body [16, 17].

On conventional radiographs, rounded or ovoid radiopaque densities, particularly in a linear or stacked configuration, should quickly raise concern for multiple magnets. Antidependent or atypical locations may also be a clue of nonmobile magnets attracted to one another.

In our case, both of these factors were evident and allowed for timely diagnosis and management. Nearly identical imaging features were shown by Brown et al. and Solis et al. in the two other reported cases of epiglottic magnetic foreign bodies [18, 19], suggesting that although unusual, the radiographic findings are essentially pathognomonic for adherent magnets at this location.

Mucosal injury and pressure necrosis of the epiglottis from magnetic attraction could occur relatively rapidly (possibly as soon as 13 hours postingestion), as demonstrated in the previous case reports [18, 19]. As such, clinicians must operate under the premise that “time is tissue.” We recommend prompt communication of this critical result to the most responsible physician, as well as liaison with the pediatric otolaryngology team to streamline potential intervention.

Due to their risk for serious injury, there has been multiple recalls issued by the United States Consumer Product Safety Commission (CPSC) for some of the once-popular neodymium magnet toys, such as Buckyballs and Magtastik [20–22]. The CPSC also issued a Safety Standard for Magnet Sets in 2012, with limits on the size and flux of small rare-earth magnets [23]. However, after multiple appeals and hearings, mandatory recall for products such as Zen Magnets have been recently reversed in 2018, and the incidence of magnetic foreign body ingestions may see an upward trend [24].

## 4. Conclusion

Rare-earth magnet apposition across the epiglottis is an unusual event but its imaging findings are unique, allowing for expedited diagnosis and management. A child presenting with a history of magnetic foreign body ingestion should be prompted triaged and investigated via clinical and imaging means. Early recognition of these spherical metallic bodies is essential for optimizing patient outcomes and preventing pressure necrosis.
